# Overexpression of *UGT74E2*, an Arabidopsis IBA Glycosyltransferase, Enhances Seed Germination and Modulates Stress Tolerance via ABA Signaling in Rice

**DOI:** 10.3390/ijms21197239

**Published:** 2020-09-30

**Authors:** Ting Wang, Pan Li, Tianjiao Mu, Guangrui Dong, Chengchao Zheng, Shanghui Jin, Tingting Chen, Bingkai Hou, Yanjie Li

**Affiliations:** 1 The Key Laboratory of Plant Development and Environment Adaptation Biology, Ministry of Education, School of Life Science, Shandong University, Qingdao 266237, China; wangting0902@mail.sdu.edu.cn (T.W.); 201912290@mail.sdu.edu.cn (T.M.); 201920299@mail.sdu.edu.cn (G.D.); chentingting87@sdu.edu.cn (T.C.); bkhou@sdu.edu.cn (B.H.); 2 College of Pharmacy, Liaocheng University, Liaocheng 252000, China; lipan@lcu.edu.cn; 3 State Key Laboratory of Crop Biology, College of Life Sciences, Shandong Agricultural University, Taian 271018, China; cczheng@sdau.edu.cn; 4 School of Life Science, Qingdao Agricultural University, Qingdao 266109, China; shanghuijin@qau.edu.cn

**Keywords:** UDP-glycosyltransferases, glycosylation, indole-3-butyric acid, abscisic acid, drought, salt

## Abstract

UDP-glycosyltransferases (UGTs) play key roles in modulating plant development and responses to environmental challenges. Previous research reported that the Arabidopsis UDP-glucosyltransferase 74E2 (*AtUGT74E2*), which transfers glucose to indole-3-butyric acid (IBA), is involved in regulating plant architecture and stress responses. Here, we show novel and distinct roles of *UGT74E2* in rice. We found that overexpression of *AtUGT74E2* in rice could enhance seed germination. This effect was also observed in the presence of IBA and abscisic acid (ABA), as well as salt and drought stresses. Further investigation indicated that the overexpression lines had lower levels of free IBA and ABA compared to wild-type plants. Auxin signaling pathway gene expression such as for *OsARF* and *OsGH3* genes, as well as ABA signaling pathway genes *OsABI3* and *OsABI5,* was substantially downregulated in germinating seeds of *UGT74E2* overexpression lines. Consistently, due to reduced IBA and ABA levels, the established seedlings were less tolerant to drought and salt stresses. The regulation of rice seed germination and stress tolerance could be attributed to IBA and ABA level alterations, as well as modulation of the auxin/ABA signaling pathways by *UGT74E2*. The distinct roles of *UGT74E2* in rice implied that complex and different molecular regulation networks exist between Arabidopsis and rice.

## 1. Introduction

Seed germination is a critical process during the plant lifecycle, which influences the yield of direct seeding in plants. For crop plants, fast germination and high germination rates are crucial agricultural traits that lead to better growth and high yield. Seed germination can be influenced by many external factors, such as temperature, water availability, light, oxygen, stress conditions, etc. Inner cues, particularly phytohormones, also behave as key regulators to determine seed dormancy and germination. Of these, abscisic acid (ABA) and gibberellins (GAs) are well-investigated plant hormones regarding the control of seed dormancy and germination. Gibberellins (GAs) contribute to break seed dormancy and promote germination [1], and several other hormones, including brassinosteroids, ethylene, and cytokinin, show the same effects in enhancing seed germination [2,3]. However, ABA maintains seed dormancy and inhibits seed germination, which are antagonistic effects against the hormones listed above. Many mutants of ABA biosynthesis genes are nondormant or show reduced dormancy, e.g., *nced6 nced9* double mutants show reduced seed dormancy [4] and *aba3-2* clearly shows a nondormant phenotype [5]. The disruption of ABA catabolism genes, such as *CYP707A1*, *CYP707A2*, and *CYP707A3,* exhibit enhanced seed dormancy [6,7].

Auxin is a critical phytohormone which mediates diverse developmental responses in plants, such as adventitious root initiation, apical dominance, vascular tissue formation, as well as flower and fruit development [6,7]. Previous studies also reported the involvement of auxin in maintaining seed dormancy and inhibiting seed germination. Liu et al. (2013) found that in Arabidopsis, exogenous application of indole-3-acetic acid (IAA) effectively enhanced seed dormancy in a dose-dependent manner [8]. There is no doubt that this process is closely related to auxin homeostasis mediated by auxin biosynthesis, degradation, transport, and conjugate modification [9], which also affects auxin signaling [10]. Consistently, it was also found that disruption of IAA biosynthesis genes *YUC1* and *YUC6* greatly enhanced seed germination [8]. The *arf10arf16* double mutant also showed better germination than wilde type (WT) [8]. A few studies also clarified the elaborate signaling networks and the sophisticated crosstalk occurring between auxin and ABA in regulating a series of developmental processes. Brady et al. (2002) reported that *ABI3* is involved in auxin signaling and regulates lateral root development [11]. Shuai et al. (2017) found that exogenous auxin application could repress soybean seed germination by decreasing the gibberellin/abscisic acid (GA/ABA) ratio [12]. A recent study reported that, to prevent the repressive effect of ABA on fruit growth, GA and auxin suppressed ABA accumulation via activation of the expression of a cytochrome P450 monooxygenase that catalyzes ABA catabolism during fruit ripening [13].

Glycosylation is an important way to buffer the homeostasis of phytohormones. Over the past decades, a few auxin-related UDP-glucosyltransferases (UGTs) were identified in a few plants to play crucial roles in regulating plant development and stress responses. For instance, *iaglu* was the first IAA glycosyltransferase identified in maize [14]. The homolog of iaglu was recently identified in rice, and was shown to be involved in catalyzing IAA glycosylation and regulating seed vigor [15]. In Arabidopsis, a few auxin-related UGTs were also identified, such as *UGT84B1* in IAA catalysis, *UGT74E2* activity toward indole-3-butyric acid (IBA), and *UGT74D1* in IAA and IBA modification [16,17,18]. Recently, Chen (2020) found that *UGT76F1* was involved in regulating auxin homeostasis by glycosylation of indole-3-pyruvic acid (IPyA), a major precursor of the auxin indole-3-acetic acid (IAA) biosynthesis, which mediates auxin-dependent hypocotyl elongation in Arabidopsis [19]. Interestingly, several auxin-related UGTs were found to be involved in regulating both plant development and stress responses. It was reported that *UGT74E2* catalyzes the glycosylation of IBA and also participates in modulating plant architecture and water deficit stress tolerance [17]. Additionally, Arabidopsis *UGT75D1* was found to affect seed germination under stress conditions, likely by regulating ARF16–ABI3 signaling [20].

In this study, in an attempt to generate transgenic rice tolerant to water deficit, we introduced *UGT74E2* into rice. However, after analyzing the transgenic plants, we found that *UGT74E2* caused effects in rice distinct from those observed in Arabidopsis. Overexpression of *UGT74E2* promoted seed germination in the presence of IBA and ABA, and also under abiotic stress conditions. Further investigation indicated that the overexpression lines had lower levels of free IBA and ABA compared to wild-type plants. Auxin signaling pathway gene expression, such as for *OsARF* and *OsGH3* genes and ABA signaling pathway genes *OsABI3* and *OsABI5,* was substantially downregulated in germinating seeds of *UGT74E2* overexpression lines. The regulation of seed germination and stress tolerance by *UGT74E2* may occur due to modulation of IBA and ABA levels and signaling pathways. The distinct roles of *UGT74E2* in rice implied that complex and different molecular regulation networks exist between Arabidopsis and rice.

## 2. Results

### 2.1. UGT74E2 Catalyzes IBA Glycosylation and Enhances Seed Germination in Transgenic Rice

**Figure 1:**
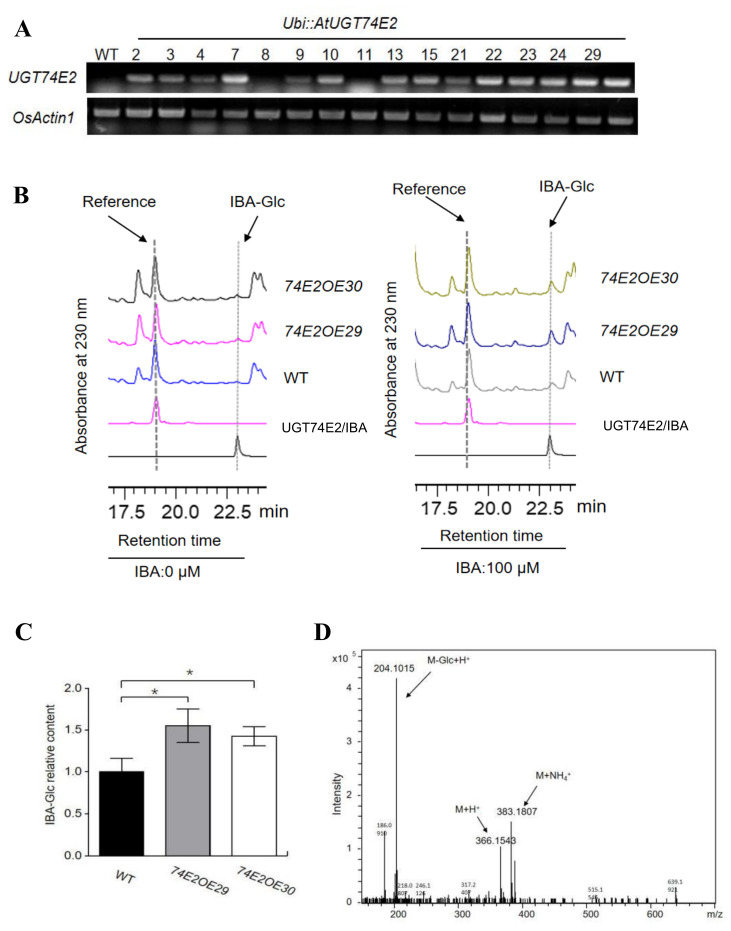
*UGT74E2* catalyzes indole-3-butyric acid (IBA) glycosylation in rice. (**A**) RT-PCR analysis of the mRNA levels of *UGT74E2* in transgenic rice plants. (**B**) HPLC profiling of IBA glucose conjugates (IBA-Glc) extracted from seven-day-old wild type (WT) and *UGT74E2*-overexpressed lines after 0 μM IBA or 100 μM IBA treatment for 12 h. (**C**) Relative IBA-Glc contents in *UGT74E2* transgenic rice plants (* *p* < 0.05). (**D**) LC-MS confirmation of IBA-glucose ester under positive ion mode.

To explore the effect of *UGT74E2* in rice plant development, we generated transgenic rice lines overexpressing Arabidopsis *UGT74E2*. In total, we obtained 14 *UGT74E2* transgenic lines. Based on the relatively high expression level of *UGT74E2* assessed by RT-PCR, OE29 and OE30 were selected for further analysis (Figure 1A). We firstly evaluated the role of *UGT74E2* in catalyzing substrate IBA in rice. Under nontreatment conditions, inner IBA-glucose ester (IBA-Glc) levels were very low and beyond detection in WT and *UGT74E2*-overexpressed plants (Figure 1B). When applying exogenous IBA to the plants, the level of IBA-Glc was strongly increased, and the relative contents of IBA-Glc in the OE29 and OE30 transgenic lines were much higher than that in WT (Figure 1B,C). The peak of IBA-Glc separated by HPLC analysis was further confirmed by LC-MS (Figure 1D). The biochemical assay verified the role of *UGT74E2* in catalyzing IBA glycosylation in rice.

### 2.2. UGT74E2 Promotes Seed Germination and Shoot Growth in the Presence of IBA and ABA

**Figure 2:**
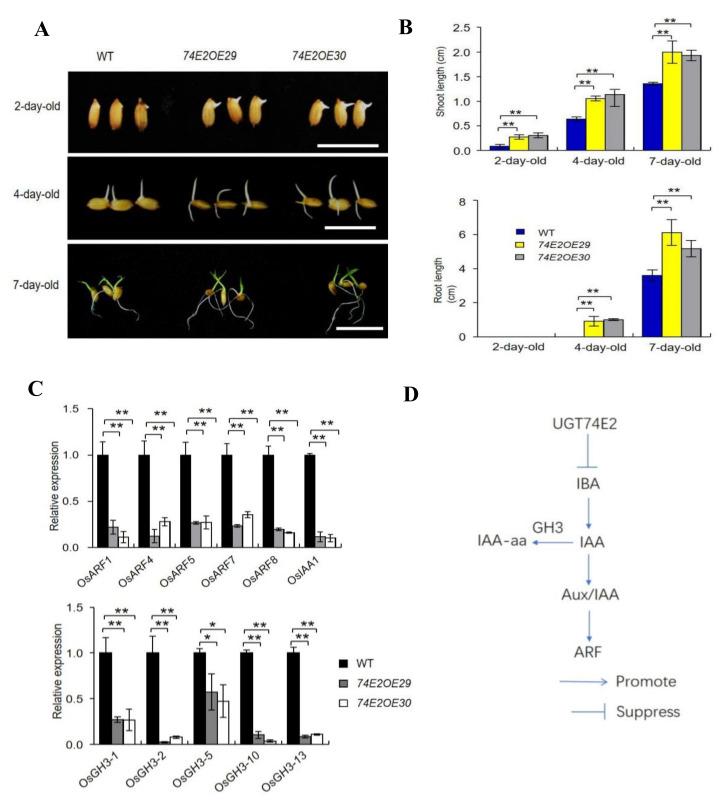
*UGT74E2* affected the seed germination and postgermination growth of the transgenic plants. (**A**) Seed germination and postgermination growth of *UGT74E2* transgenic plants. Here, at least 30 seeds were observed for each line (bars = 3 cm). (**B**) Shoot and root lengths of *UGT74E2* overexpression lines for 2, 4, and 7 days. Bars represent standard deviation of at least 10 seedlings. (**C**) Expression of auxin signaling pathway genes in seven-day-old *UGT74E2*-overexpressed rice plants compared to WT. Transcript levels were normalized to the mRNA levels of *OsActin*. Data shown are means ± SD. Student’s *t*-test was performed (* *p* < 0.05, ** *p* < 0.01). Experiments were conducted for three biological replicates. (**D**) Location of *GH3*, *IAA,* and *ARF* genes in the auxin biosynthesis and signaling pathway.

In analyzing the biological roles of *UGT74E2* in rice, we firstly noticed that overexpression of *UGT74E2* resulted in faster seed germination, which also accelerated subsequent seedling growth (Figure 2A,B). This result suggests that *UGT74E2* might play important roles in the regulation of seed germination and postgermination growth by glycosylating IBA in rice. Additionally, to see whether the auxin signaling pathway was affected, we detected some crucial genes involved in the auxin signaling pathway in seven-day-old seedlings, including *OsARF1, OsARF4, OsARF5, OsARF7, OsARF8* [21], the transcription repressor *OsIAA1* [7], as well as auxin amido synthetase genes *OsGH3-1, OsGH3-2, OsGH3-5, OsGH3-10, and OsGH3-13* (Figure 2D), which help to maintain auxin homeostasis [22]. qRT-PCR results showed that all of these genes were significantly downregulated in the overexpression plants, demonstrating that auxin signaling and homeostasis was perturbed in rice upon *UGT74E2* overexpression (Figure 2C).

**Figure 3:**
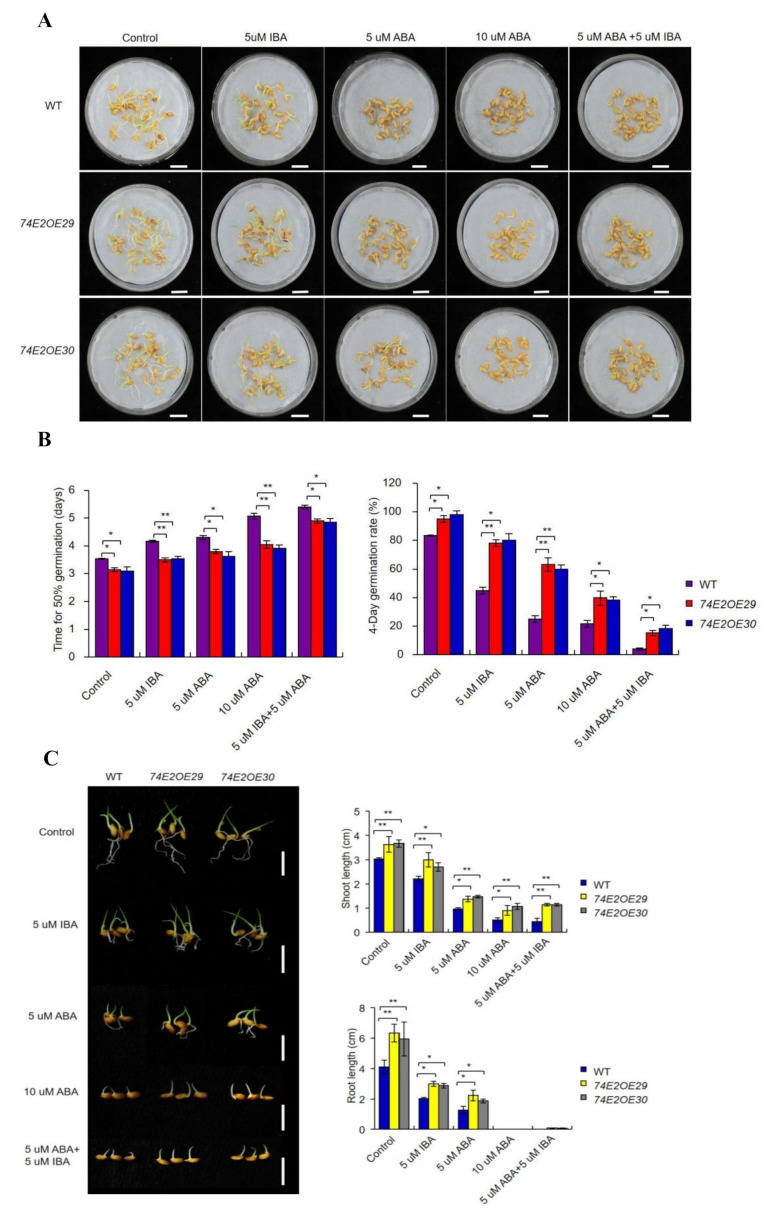
*UGT74E2*-overexpressed plants are not sensitive to IBA and abscisic acid (ABA) treatment. (**A**) Seed germination of WT and *UGT74E2* transgenic rice under IBA and ABA treatments for eight days. Here, at least 30 seeds were observed for each line (bars = 3 cm). (**B**) Time to 50% germination and four-day germination rates were calculated in the presence of IBA and ABA. (**C**) Shoot and root lengths of *UGT74E2-*overexpressed lines in the presence of IBA and ABA (scale bars = 3 cm). Experiments were conducted for three biological replicates. Bars represent standard deviation of at least 10 seedlings. Student’s *t*-test was performed (* *p* < 0.05, ** *p* < 0.01).

Previous studies reported that auxin induces seed dormancy and represses seed germination [8,12]. In our study, we treated the WT, OE29, and OE30 seeds with IBA, and observed that *UGT74E2* promoted seed germination and shoot growth in OE29 and OE30 lines in the presence of IBA (Figure 3A–C). In comparison, WT was much more delayed in seed germination and seedling growth. Abscisic acid (ABA), as the major endogenous factor, is also well studied regarding seed germination repression and postgermination growth [23]. Thus, to see whether *UGT74E2*-overexpressed lines respond to ABA, we also subjected the WT, OE29, and OE30 seeds to ABA treatment (Figure 3A–C). Seed germination and shoot growth of OE29 and OE30 were observed to be more accelerated than in WT (Figure 3A–C), meaning that they are not sensitive to ABA. In a recent study, He et al. (2020) examined the interaction between IAA and ABA, and found that exogenous IAA enhanced ABA-mediated reduction of seed vigor in rice [15]. Accordingly, we also observed that the combination of IBA and ABA suppressed seed germination more strongly than a single hormone (Figure 3A–C). In the presence of both hormones, seed germination and shoot growth were obviously promoted by *UGT74E2* overexpression compared to WT. These observations imply that the effect of exogenous IBA and ABA on seed germination in OE29 and OE30 could be alleviated by *UGT74E2*.

### 2.3. UGT74E2 Overexpression Affects Auxin and ABA Response

**Figure 4:**
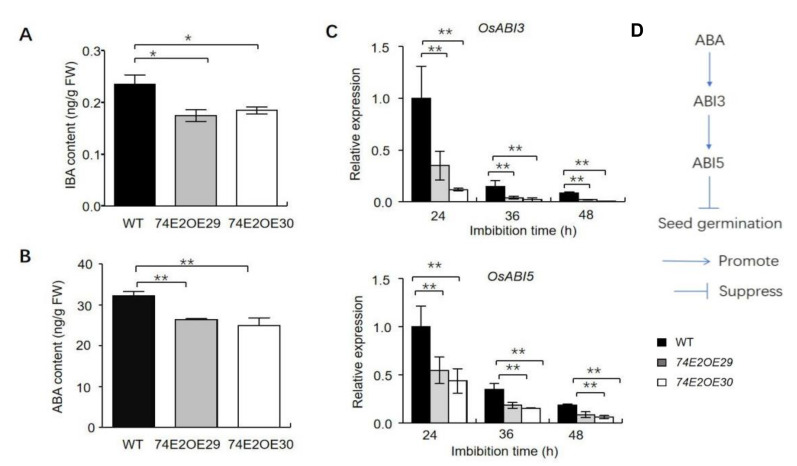
*UGT74E2* altered IBA and ABA contents in germinating rice seeds. (**A**) The contents of IBA in germinating WT and *UGT74E2* transgenic rice seeds after being imbibed for 24 h. (**B**) The contents of ABA in germinating WT and *UGT74E2* transgenic rice seeds after being imbibed for 24 h. (**C**) Expression of *OsABI3* and *OsABI5* in germinating seeds in WT and *UGT74E2* transgenic rice. Gene expression was normalized to the reference gene *OsActin*. For each experiment, three replicates were done; data are presented as means ± SD. Student’s *t*-test for each genotype was performed (* *p* < 0.05, ** *p* < 0.01). (**D**) Role of *ABI3* and *ABI5* genes in the ABA signaling pathway during seed germination.

Glycosyltransferases can regulate auxin and ABA homeostasis in plants [24,25]. To further understand *UGT74E2* function, the levels of IBA and ABA were assessed after 24 h of seed imbibition. IBA and ABA levels were significantly decreased in germinating seeds in *UGT74E2*-overexpressed lines compared to in WT plants (Figure 4A,B). Furthermore, ABA signaling pathway gene expression of *ABI3* and *ABI5* was also detected by qRT-PCR after seed imbibition for 24, 36, and 48 h, respectively (Figure 4C,D). Consistently, the relative mRNA levels of *ABI3* and *ABI5* were found to be dramatically downregulated in the *UGT74E2*-overexpressed lines compared to wild type, largely contributing to break seed dormancy and promote seed germination in *UGT4E2*-overexpressed lines. Since GA is also a key regulator in seed germination and plays antagonistic roles with ABA [26], we also evaluated the expression of several genes involved in GA biosynthesis in imbibed seeds, including *GA3ox2*, *OsGA20ox1*, *OsGA20ox2,* and *OsKO2*. The results showed that all four genes were upregulated in *UGT74E2*-overexpressed lines (Figure S1), thereby enhancing GA biosynthesis. This finding, in another aspect, supported our above observation that ABA levels and signaling declined upon *UGT74E2* overexpression.

### 2.4. UGT74E2 Overexpression in Rice Showed Enhanced Seed Germination and Shoot Elongation under Abiotic Stress

**Figure 5:**
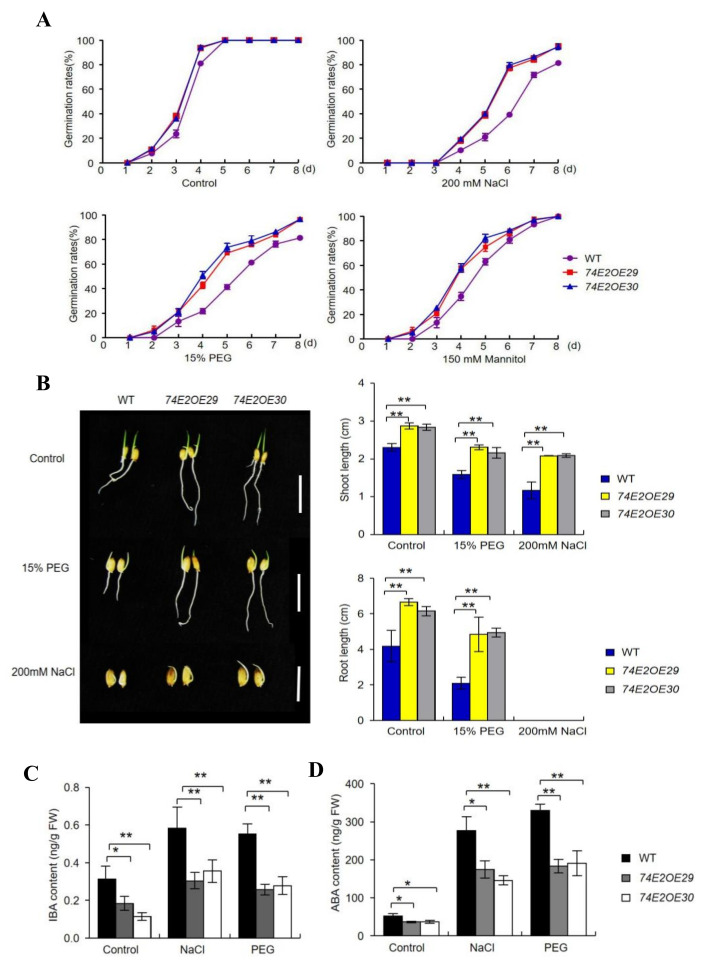
*UGT74E2* transgenic lines are not sensitive to salt and drought stresses during the germination and postgermination stagea. (**A**) Germination rates of WT and *UGT74E2*-overexpressed lines under NaCl, polyethylene glycol (PEG), and mannitol treatment. (**B**) Shoot and root lengths of *UGT74E2*-overexpressed lines under PEG and NaCl treatment (scale bars = 3 cm). (**C**) IBA and (**D**) ABA contents in seven-day-old rice after 200 mM NaCl and 15% PEG treatment for 12 h. Data are means ± SD. For each experiment, three biological replicates were done. Student’s *t*-test was performed (* *p* < 0.05, ** *p* < 0.01).

ABA and auxin play important roles in plant responses to drought and salt stresses. To study the function of *UGT74E2* in abiotic stress responses, we treated OE29 and OE30 with NaCl and polyethylene glycol (PEG), respectively, and recorded the seed germination rates after eight days (Figure 5A). Under nontreatment conditions, the germination rates of *UGT74E2OE* seeds were higher than those of wild type. Upon exposure to 200 mM NaCl, 15% PEG, and 150 mM mannitol, the germination of WT seeds was largely inhibited, while OE29 and OE30 seeds were less affected. After six days, nearly 80% of *UGT74E2OE* seeds germinated under 200 mM NaCl treatment, and 40% of WT. When subjected to 15% PEG, approximately 80% *UGT74E2OE* seeds germinated but only 60% WT seeds germinated. In the presence of 150 mM mannitol, the germination rates of *UGT74E2OE* lines were approximately 90%, while less than 80% WT seeds germinated. On the tenth day, we measured shoot and root elongation under different conditions and found that the shoots and roots of the *UGT74E2OE* plants were longer than those of WT plants under NaCl, PEG. and mannitol treatments (Figure 5B), suggesting that overexpression of *UGT74E2* enhanced seed germination and postgermination growth in rice under drought and salt stresses. IBA and ABA level were further measured in WT and *UGT74E2*-overexpressed lines, with both hormones found to be significantly increased after stress treatment, indicating that they are important players involved in stress responses. In comparison with WT, IBA and ABA were observed to accumulate less in the *UGT74E2*-overexpressed lines under both control and stress conditions (Figure 5C,D), further demonstrating that *UGT74E2* reduces IBA levels by glycosylation and also influences ABA levels.

### 2.5. Established UGT74E2-Overexpressed Seedlings Are Sensitive to Abiotic Stresses

**Figure 6:**
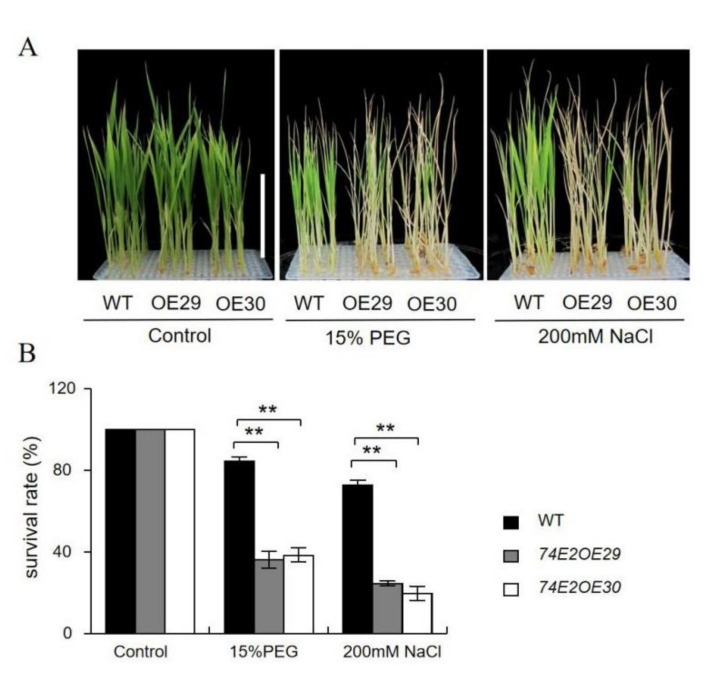
*UGT74E2*-overexpressed lines are less tolerant to polyethylene glycol (PEG) and NaCl during the seedling stage. The growth (**A**) and survival rates (**B**) of *UGT74E2* transgenic rice plants upon exposure to 200 mM NaCl and 15% PEG for seven days (scale bars = 10 cm). For each experiment, three biological replicates were performed. Student’s *t-*test was performed (** *p* < 0.01).

Next, we tested the growth of established seedlings under drought and salt stresses. The WT, OE29, and OE30 lines were kept under normal conditions in nutrient solution for three weeks to grow, then treated with 200 mM NaCl and 15% PEG, respectively, for one more week. Interestingly, the *UGT74E2-*overexpressed lines showed sensitivity to NaCl and PEG treatments. OE29 and OE30 were observed to turn yellow and became more withered compared to WT seedlings (Figure 6A); the survival rates of OE29, OE30, and WT were 28.3%, 26.6%, and 78.3%, respectively, under NaCl treatment, and 35.6%, 36.8%, and 81.5%, respectively, under PEG treatment (Figure 6B), demonstrating that the stress response regulated by *UGT74E2* is highly dependent on ABA. Undoubtedly, the reduced ABA levels in the *UGT74E2*-overexpressed lines (Figure 5D) largely accounted for the sensitive phenotype under abiotic stress conditions.

### 2.6. Overexpression of UGT74E2 Leads to Increased Reactive Oxygen Species Accumulation

**Figure 7:**
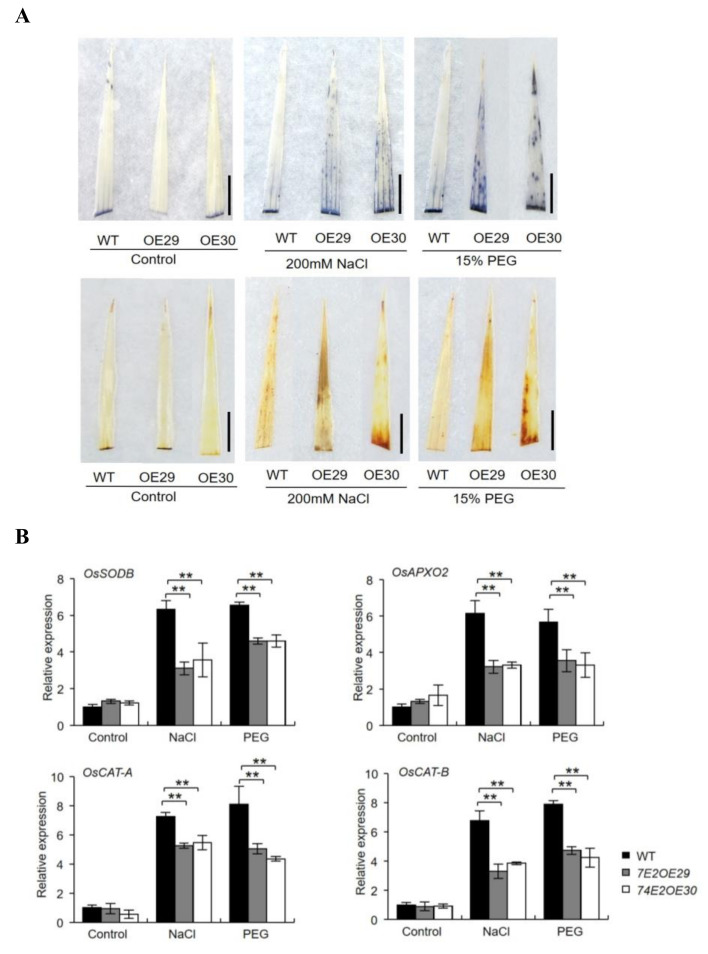
Accumulation of ROS under NaCl and PEG treatments. (**A**) Two-week-old seedlings were subjected to nitroblue tetrazoliun (NBT) and diaminobenzidine (DAB) staining after treatment with NaCl and polyethylene glycol (PEG) for 24 h. (scale bars = 1 cm) (**B**) Genes encoding antioxidant enzymes are downregulated upon *UGT74E2* overexpression under abiotic stress. For RNA extraction, seven-day-old rice plants were subjected to 200 mM NaCl and 15% PEG treatment for 12 h. Gene expression was normalized to the reference gene *OsActin*. For each experiment, three replicates were done; data are presented as means ± SD. Student’s *t*-test for each genotype was performed (** *p* < 0.01).

Abiotic stress tends to result in overproduction of ROS and causes oxidative damage to the plants. IBA levels could also affect ROS accumulation in plants [12]. Thus, we performed nitroblue tetrazoliun (NBT) and diaminobenzidine (DAB) staining on the three-week-old, healthy WT and *UGT74E2OE* plants after they were exposed to NaCl and PEG treatments. Under nontreatment conditions, no staining was observed for the three lines. When treated with NaCl and PEG, all rice leaves were stained, and the color in OE29 and OE30 was much stronger than that in WT plants (Figure 7A), indicating that ROS accumulates more in *UGT74E2*-overexpressed plants. To explain the process at the molecular level, we also investigated the expression of antioxidant enzyme encoding genes, including *OsSODB*, *OsAPXO2*, *OsCAT-A*, and *OsCAT-B*. Consistently, upon exposure to NaCl and PEG conditions, the expression levels of these genes were more upregulated in WT than in the *UGT74E2*-overexpressed plants (Figure 7B), indicating that *UGT74E2* overexpression in rice repressed antioxidant gene expression, thereby reducing ROS scavenging capacity.

### 2.7. Expression Analysis of Stress-Responsive Genes under Drought and Salt Stresses

**Figure 8:**
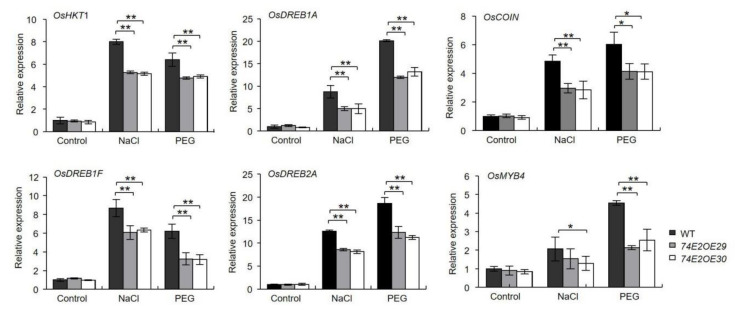
Expression of stress-responsive genes is downregulated upon *UGT74E2* overexpression under abiotic stress. For RNA extraction, seven-day-old rice plants were subjected to 200 mM NaCl and 15% PEG (polyethylene glycol) treatment for 12 h. Gene expression was normalized to the reference gene *OsActin*. For each experiment, three replicates were done; data are presented as means ± SD. Student’s *t*-test for each genotype was performed (* *p* < 0.05, ** *p* < 0.01)

To explore the possible molecular pathways of *UGT74E2* involved in regulating stress responses in rice, we evaluated the mRNA levels of four stress-responsive genes with real-time PCR, such as *OsHKT1*, *OsDREB1A*, *OsDREB1F*, *OsCOIN, OsMYB4,* and *OsDREB2A*. As shown in Figure 8, under normal conditions, no significant differences were observed. After treatment with NaCl and PEG, the expression of these stress-related genes was greatly elevated, while the upregulation of these genes in wild type was more prominent than in *UGT74E2OE* lines, implying that overexpression of *UGT74E2* could decrease the expression of stress-responsive genes when facing stress conditions, which may contribute to the reduced stress tolerance of the plant.

## 3. Discussion

Faster seed germination is an index of seed vigor, which also accelerates seedling establishment and growth. In this study, we transferred Arabidopsis *UGT74E2* into rice and investigated its role and mechanism in regulating rice seed germination and stress responses. We found that *UGT74E*2 overexpression accelerated rice seed germination (Figure 2A). This effect was also observed in the presence of IBA and ABA (Figure 3A), and also under abiotic stress conditions (Figure 5A). Our findings were highly consistent with a recent study [15] reporting that knockout of a rice IAA glycosyltransferase gene *IAGLU* led to lower seed vigor. The *iaglu* mutant is sensitive to exogenous application of IBA and ABA, and both IBA and ABA contents were increased in the *iaglu* mutant, further inducing the expression of *ARF* genes and *ABI* genes and leading to lower seed germination and slower postgermination growth [15]. A former study also showed that *UGT75D1*, a glycosyltransferase preferring auxin indole-3-butyric acid, plays positive roles in regulating seed germination and seedling greening under abiotic stress by modulating ARF16–ABI3 signaling [20]. Accordingly, we also found that both IBA and ABA levels declined in *UGT74E2*-overexpressed plants (Figure 4A,B), and their signaling pathway were also affected, which was supported by the downregulation of auxin signaling/biosynthesis pathway genes, such as *ARF*, *IAA,* and *GH3* genes (Figure 2C), as well as ABA signaling pathway genes, such as *ABI3* and *ABI5* (Figure 4C). All these data accounted for the fast seed germination of the *UGT74E2*-overexpressed lines. This process, modulated by *UGT74E2,* highlighted the crosstalk between the IBA and ABA signaling pathways. Both IBA and ABA contents decreased in *UGT74E2*-overexpressed plants, indicating that *UGT74E2* may be involved in modulating homeostasis of the two hormones. Auxin was previously reported to repress seed germination by decreasing the GA/ABA ratio in soybean [27]. Accordingly, we found that the GA/ABA ratio increased upon *UGT74E2* overexpression (Figure 4 and Figure S1), which accelerated seed germination. This also implies close crosstalk between ABA and GA in *UGT74E2*-overexpressed lines.

In this study, we observed that the *UGT74E2-*overexpressed lines exhibited enhanced germination and postgermination growth under abiotic stress conditions (Figure 5A,B), however, when exposing the established seedlings to PEG and NaCl, they showed sensitivity to these treatments (Figure 6A,B). These findings indicate that the *UGE74E2*-mediated stress response is highly dependent on the ABA signaling pathway. Similar effects were also observed for many other genes involved in the ABA-dependent pathway, such as *DREB* transcription factors [28,29], the cucumber *ATAF1* gene [30], the Arabidopsis *UGT76C2* gene, etc. [31]. When the germinating seeds were exposed to water deficit conditions such as drought and salinity, ABA was quickly synthesized and induced ABI3 and ABI5 expression, which then inhibited seed germination and arrested postgermination growth [32,33]. The process is actually a protective mechanism to avoid harm to young seedlings [34,35]. In *UGT74E2*-overexpressed lines, endogenous ABA levels were decreased compared to WT plants (Figure 4B), therefore, the overexpression (OE) lines exhibited enhanced germination and postgermination growth in response to drought and salinity (Figure 5A,B). However, after transition into the seedling stage, an opposite response was observed for the *UGT74E2*-overexpressed lines, which showed stress sensitivity to PEG and NaCl treatment in comparison to WT (Figure 6A,B). It is well known that ABA accumulates in response to drought and salt stresses and plays critical roles in responding to stress; the reduced ABA levels observed in our study undoubtedly weakened the stress response in *UGT74E2-*overexpressed lines (Figure 5D). Supportive to this view, genes contributing to ABA production play positive roles in plant adaptations to abiotic stresses. For instance, overexpression of *OsNCED3*, a key rate-limiting enzyme in rice enhances abiotic stress tolerance in rice, whereas knockout of this gene results in a stress-sensitive phenotype [34]. HAT1 and HAT3, two class II HD-ZIP transcription factors, were identified as negative regulators of ABA biosynthesis. Plants overexpressing *HAT1* exhibit ABA-insensitive phenotype and are less tolerant to drought stress, while *hat1hat3* double mutants show enhanced tolerance to drought stress [35].

Inner auxin levels were reported to be closely related to plant stress adaptations [36]. Park et al. (2013) reported that the transgenic potato overexpressing *AtYUC6* exhibited high auxin levels and reduced ROS accumulation, thus enhancing the drought-tolerant phenotype [36]. Shi et al. (2014) found that the iaaM-OX transgenic lines with higher endogenous indole-3-acetic acid (IAA) levels exhibited enhanced drought stress resistance, while yuc1yuc2yuc6 triple mutants with lower endogenous IAA levels showed decreased stress resistance in comparison to nontreated WT plants [37]. Moreover, exogenous application of IAA was also shown to reduce H_2_O_2_ and O_2_^-^ levels in response to abiotic stresses, as well as lead to higher activities of antioxidant enzymes to cope with abiotic stresses [30]. In agreement with this point, we found that the *UGT74E2*-overexpressed plants showed significantly higher ROS production under drought and salt treatment (Figure 7A), suggesting that ROS accumulation might be enhanced via endogenous IBA level reduction and elevated expression of antioxidant enzyme genes upon *UGT74E2* overexpression (Figure 7B). In addition, auxin was reported to induce the expression of many abiotic stress-related genes, such as *RAB18, RD22, RD29A, RD29B, DREB2A,* and *DREB2B*, thereby contributing to improved abiotic stress resistance [30]. Consistently, in our study, *UGT74E2* overexpression repressed some abiotic stress-responsive genes in rice, including *OsHKT1*, *OsDREB1A*, *OsDREB1F*, *OsCOIN, OsMYB4,* and *OsDREB2A* (Figure 8), which, to some degree, explained the sensitivity of *UGT74E2OE* seedlings under confronting stress.

Previous research reported that *UGT74E2OE* lines exhibited increased IBA-glc as well as free IBA content, with the explanation being that increasing IBA-glucosyltransferase activity could induce IBA synthesis [17]. However, our study found that overexpression of *UGT74E2* in rice led to decreased free IBA (Figure 4A) and increased IBA-glc levels (Figure 1B,C). The distinct effects caused by *UGT74E2* in rice and Arabidopsis underlined that the complex regulation of auxin homeostasis exists in different plants, and *UGT74E2* is likely to be involved in different cues in rice and Arabidopsis. However, our observations were consistent with a few former studies. Liu et al. (2019) reported that ectopic expression of *OsIAGT1* led to declined endogenous IAA content and increased levels of IAA-Glc [38]. The induction of *UGT74D1* increased the level of OxIAA-Glc and decreased OxIAA remarkably [18]. We also analyzed the expression of auxin signaling pathway genes in *UGT74E2*-overexpressed rice. In line with the reduced IBA content, expression of auxin signaling pathway genes was reduced in OE29 and OE30 rice plants (Figure 2C). These results further confirmed that *UGT74E2* negatively affects auxin levels and signaling in rice, which is different from what is observed in Arabidopsis.

In addition, it was also reported that overexpression of *UGT74E2* in Arabidopsis enhances stress responses [17]. However, we found that *UGT74E2-*overexpressed plants are sensitive to salt and drought stress at the seeding stage. These observations demonstrate that a gene can acquire new functions or cause distinct effects via heterologous expression in other species. Some literature also supports this view. For instance, Quilis et al. (2008) found that transgenic rice overexpressing Arabidopsis *NPR1* showed sensitivity to drought and salt stress [39]. However, overexpression of *AtNPR1* in tobacco enhanced oxidative stress tolerance in transgenic plants [40]. *AtBBX21* could promote seed germination and seedling photomorphogenesis in Arabidopsis [41,42]. Heterologous expression of *AtBBX21* in potato was more robust, resulted in more tuber production, and showed higher rates of photosynthesis [43]. Moreover, *AtBBX32*, as a member of the B-box gene family, could regulate light signal transduction in Arabidopsis [44,45], while increasing grain yield in soybean [45]. The novel roles of the gene acquired by heterologous expression imply complex molecular regulation networks in different plant species. Heterologous expression is worth further exploration, as novel and valuable traits might be generated by introducing this gene into other plants.

## 4. Materials and Methods

### 4.1. Cloning and Plasmid Construction of UGT74E2

To generate *UGT74E2*-overexpressed lines, a full-length *UGT74E2* cDNA coding sequence was cloned from *Arabidopsis thaliana* into a pUN1301 binary vector, which was driven by a ubiquitin promoter. Rice transformation was performed by Biorun biological company (Wuhan, China, http://www.biorun.net/).

### 4.2. Seed Germination

Thirty seeds per replicate of wild type (WT) and *UGT74E2*-overexpressed lines were imbibed in 9-cm diameter Petri dishes with 10 mL distilled water at 28 °C for 8 days. Seed germination of wild type and *UGT74E2*-overexpressed lines was also conducted under various concentrations of IBA, ABA, salt, and PEG treatments. The germination rates of the seeds were tested. Three replications were performed.

### 4.3. Plant Materials and Stress Treatment

The rice seeds were surface sterilized in 75% ethanol (*v*/*v*) for 2 min, then 0.1% mercuric chloride solution for 3 min, and finally washed 3–4 times with deionized water. For salt treatment in nutrient solution, plants were grown in nutrient solution for three weeks at 28 °C, followed by treatment with 200 mM saline or water (control plants) for one more week. At least 60 plants per line were assayed. After salt treatment, the percentage of surviving plants was determined. For drought stress treatment in nutrient solution, plants were grown in nutrient solution for three weeks under normal conditions. The plants were treated with 15% PEG for one more week. Plant phenotypes were observed and photographed with a digital camera (Alpha 6400, Sony, Japan).

### 4.4. HPLC and LC-MS Analysis

Reverse-phase high performance liquid chromatography (HPLC) was carried out on a Shimadzu HPLC system (Shimadzu, Japan). Samples of 20 µL each were loaded using an autosampler SIL-20A (Prominence SIL-20A) onto a 5 μm C18 column (150 mm × 4.6 mm; Welch, Ultimate, Shanghai). A linear gradient with increasing acetonitrile (solvent A) against double-distilled H_2_O (solvent B) at a flow rate of 1 mL/min over 40 min was used. Both solutions contained 0.1% H_3_PO_4_. Each peak on the chromatogram was monitored between 190 nm and 430 nm.

For liquid chromatography–mass spectrometry (LC-MS) analysis (Shimadzu, Japan), the methods and mobile phases were similar to HPLC conditions, except that 0.1% acetic acid was used instead of 0.1% trifluoroacetic acid. The mass spectrometer was operated in positive electrospray ionization mode with 50 eV and a probe voltage of 5.0 kV. The dry heater was set to 180 °C. Data acquisition and analysis were performed with Xcalibur software (v. 2.0.6) [46].

### 4.5. Determination of IBA and ABA Concentrations

To determine the IBA and ABA concentrations, 0.1 g fresh leaves of different lines were weighed and 900 μL PBS (pH 7.2–7.4) was added, followed by homogenization and centrifugation for 20 min at 3000× rpm. The supernatant was then collected. To quantify ABA, the plant hormone abscisic acid (ABA) ELISA Kit (MM-3274201, http://www.mmbio.cn/) was used, and the concentrations of ABA in the samples were determined using a microplate reader (Tecan, Infinite^TM^M200 PRO, Sursee, Switzerland). To quantify IBA, the plant hormone IBA Kit (MM-3585201, http://www.mmbio.cn/) was used, and the concentrations of IBA in the samples were determined using a microplate reader (Tecan, Infinite TMM200 PRO).

### 4.6. Detection of H2O2 and Superoxide by Diaminobenzidine (DAB) and Nitrobluetetrazolium (NBT) Staining

Three-week-old plants were treated with 200 mM NaCl and 15% PEG for 24 h. The plant leaves of each line were soaked in DAB and NBT stains for 12 h. The samples were decolorized using 95% ethanol for 1 h, and then decolorized using 75% ethanol at 70 °C. The methods of H_2_O_2_ detection and DAB staining were described by Du et al. [47]. The method of NBT staining was described by Wang et al. [48].

### 4.7. qRT-PCR

To measure the expression of relative genes by real-time PCR (qRT-PCR), the rice leaves were sampled after PEG and NaCl treatment and frozen immediately in liquid nitrogen. Total RNA was extracted from two-week-old rice plants by Trizol reagent and cDNA was obtained by using Primescript RT reagent kit. Real-time PCR reactions were performed using the Bio-Rad real-time thermal cycling system. SYBR-Green was used to detect gene expression abundances. Data were analyzed using the Bio-Rad CFX Manager software. The gene ubiquitin was used as the inner control. Primer information is included in  Supplementary Table S1. The experiments were repeated three times and the results were analyzed.

## Supplementary Materials

Supplementary Materials can be found at https://www.mdpi.com/1422-0067/21/19/7239/s1. Figure S1. Expression of GA biosynthesis pathway genes in germinating seeds imbibed for 36 h. Table S1. Sequences of primers used in this study.

## Abbreviations

ABA	Abscisic acid
DAB	Diaminobenzidine
IBA	Indole-butyric acid
HPLC	High Performance Liquid Chromatography
NBT	Nitroblue tetrazoliun
OE	Overexpression
PEG	Polyethylene glycol
WT	Wild type
