# Supplementary material for: Overexpression of UGT74E2, an Arabidopsis IBA Glycosyltransferase, Enhances Seed Germination and Modulates Stress Tolerance via ABA Signaling in Rice
Source: Int J Mol Sci. 2020 Sep 30;21(19):7239. doi: 10.3390/ijms21197239 (PMC7582762; doi:10.3390/ijms21197239)
Supplement: Supplementary file 1 [file ijms-21-07239-s001.pdf]

International Journal of
Molecular Sciences

MDPI

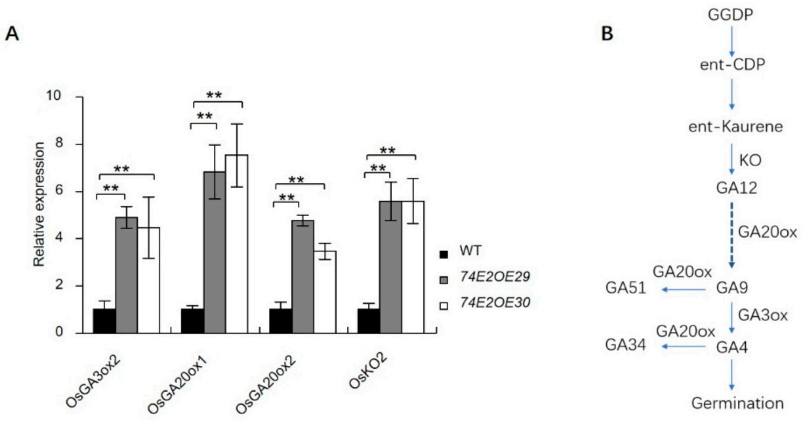

Figure S1. Expression of GA biosynthesis pathway genes in germinating seeds imbibed for 36 h (**A**). The gene expression was normalized to the reference gene *OsActin*. For each experiments, three replicates were done and data are means ± SD. Student's t test for each line was performed (* $p < 0.05$, ** $p < 0.01$). (**B**) Roles of GA biosynthesis genes in the GA biosynthesis pathway.

Table S1. Sequences of primers used in this study.

Gene	Forward (5′–3′)	Reverse (5′–3′)
	**Primers for Gene Cloning**	
UGT74E2	CGCCATATGAGAGAAGGATCTCATCTT	CCGCTCGAGACAAAACATAGAAACAAAC
	**Primers for qPCR Analysis**	
OsActin	GACCTTGCTGGGCGTGAT	GTCATAGTCCAGGGCGATGT
UGT74E2	ACTCTGGTCCTCGTCTCC	ATGGTTCCTCGCCTTC
OsABI3	TGACGATACCTTCCCGTTGC	CGAGGAGCTGGTCTATGTCG
OsABI5	AGCGGTGAACCAGTTTGATT	ATCTGCCTGTTTCCTCTCCA
OsIAA1	ATAGGTACCGAGCCGAAGCATCA	ATAGGATCCAACCACACGGTCATG
OsARF1	AGCCATCAGAATTACACCGC	ATGAACTAGCCAGTAGCCAC
OsARF4	AGACCGCATGCATCAATAGC	TCTGATTTGTTGGGCATCGG
OsARF5	CAGCAACTCCACTAATGACC	TCACCATCCAAGCTCATCTG
OsARF7	ACTACAGCGAACACAGGAAC	TATGGCCAGGTAGCATTCAG
OsARF8	ATCATGTCGGTCCAACATCC	GTCGGTTAGTATGGTAAGCC
OsGH3-1	CGGGAACAAGCAATGGAACA	CAGATCATCACCCTCTAGCTTCAA
OsGH3-2	TCATGCCCGTCATGAACTTG	TCGTCTCCGACTTGATGAACAG
OsGH3-5	GGTCGGCAAAATCTATGAAG	TGGAGGTCTTTCTCGGTGTT
OsGH3-10	AGCAGTCTCGCTACATCTGTCTTG	CGCTTTTGTATACGCTCGTCAA
OsGH3-13	TGTGTAATGTCAAACGTTGCTCAT	TGATTCATAAAGAACACTGCTCGTATT
OsDREB1A	ATGGGCTGGGACCTGTACTA	GCATCGGAAGCCAGAAAAGAG
OsMYB4	CGGTGGATCAACTACCTCCG	CGTCCAGCCTCTTCTTGAGG
OsAPxO2	TTCAGCTTTCGTTTGTGCGG	ACCACTCGCAATCCAACGAT
OsCAT-A	TTGGGGGTGAAGATTGCGAA	CGACAACAGAAGATGCGTGC
OsCAT-B	GCTTGCACAGTTTGACAGGG	CGACTGTGGAGAACCGAACA
OsSODB	ACAACGGCAACCCATTACCA	CAGCCAGACCCCAAAAGTGA
OsHKT1	GCAGCTGGAAGCAGCAATTC	AAACGAGGAGACTGTGACCG
OsDREB1F	TGTTCGACCATGGACACCGA	ACATGTCAGTCCATCCATAGCTT
OsDREB2A	ATGTATGGTCCCACAGCACG	ACAACACAGCTGGCCCATTA
OsCOIN	TCAAAGCAGCAAATGCACCC	CATGGCCATCAATGAAGCGG
OsKO2	TGCTACCAGCGACTATTGTGATTT	GTGCAGAAGTACCCAACATGCTT
OsGA20ox2	CCAATTTTGGACCCTACCGC	GAGAGAAGCCCAACCCAACC
OsGA20ox1	GCCACTACAGGGCCGACAT	TGGTTGCAGGTGACGATGAT
OsGA3ox2	TCCTCCTTCTTCTCCAAGCTCAT	GAAACTCCTCCATCACGTCACA
